# A very late paradoxical reaction occurring following antibiotic treatment of Buruli ulcer

**DOI:** 10.1371/journal.pntd.0014585

**Published:** 2026-07-28

**Authors:** Daniel P. O’Brien, Chris Mow, Jessica C. O’Keeffe

**Affiliations:** 1 Department of Infectious Diseases, Barwon Health, Geelong, Victoria, Australia; 2 Department of Infectious Diseases and Medicine, Royal Melbourne Hospital, University of Melbourne, Victoria, Australia; 3 Melbourne Pathology, Victoria, Australia; Yale School of Medicine: Yale University School of Medicine, UNITED STATES OF AMERICA

## Abstract

We report a very delayed paradoxical reaction presenting more than 4 years after antibiotic treatment of Buruli ulcer that settled without further antibiotic treatment. Our report aims to alert clinicians to this possibility to avoid misdiagnosis of relapsed infection and unnecessary antibiotic re-treatment which can be associated with toxicity, inconvenience and cost for the patient.

## Author summary

Paradoxical reactions following antibiotic treatment of Buruli ulcer are common, manifest by a worsening clinical appearance of the wound. However, they usually occur during or soon after antibiotic treatment, and recognising this usual timing helps clinicians distinguish them from treatment failure or infection relapse. Importantly, this report alerts treating clinicians that these reactions have the potential to occur many years after treatment, thereby potentially avoiding a mistaken diagnosis of disease relapse and the institution of unnecessary antibiotic re-treatment and its associated toxicity, inconvenience and cost.

## Introduction

We report a very delayed paradoxical reaction presenting more than 4 years after antibiotic treatment of Buruli ulcer that settled without further antibiotic treatment.

## Methods and results

### Ethics statement

Collection of the data described was approved by the Barwon Health Human Research and Ethics Committee. Written formal consent for publication was obtained from the person described in this manuscript.

A 61 year old previously well female with no known immune suppression, presented in December 2019 with a WHO category 3 Buruli ulcer (BU) with two separate lesions on her right ankle (Fig 1). One was an ulcerated lesion (35 mm x 20 mm diameter) in the Achilles region and the other was a non-ulcerated nodular lesion (35 mm x 22 mm diameter) on the lateral aspect. The diagnosis was confirmed by a swab of her ulcerated lesion which was *M. ulcerans* polymerase chain reaction *(*PCR) positive. Mycobacterial cultures were not performed.

**Fig 1:**
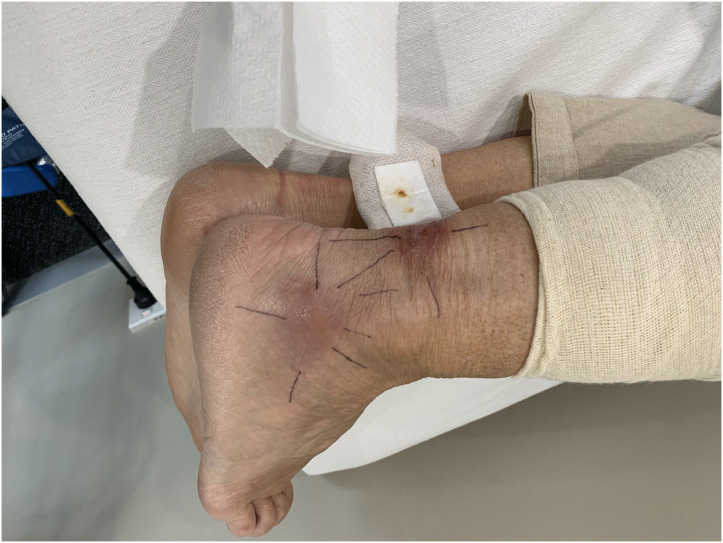
Photograph showing two Buruli ulcer lesions on the right ankle region at initial presentation; an ulcerated lesion in the Achilles region and a non-ulcerated lesion on the lateral aspect. Skin ink markings indicate the extent of induration associated with each lesion.

She was commenced on Rifampicin 300 mg twice daily and clarithromycin 500 mg twice daily but only received 49-days of a planned 56-day course as treatment was ceased early due to intolerance (nausea, headaches, dizziness, malaise). By 52 days post antibiotic commencement, the ulcerated wound had fully healed and the induration associated with the nodular lesion settled (although it never ulcerated).

The patient developed recurrent painful swelling of the nodular lesion on the lateral aspect of the ankle 109-days post antibiotic commencement (April 2020). This was diagnosed clinically as a paradoxical reaction and managed with topical corticosteroid cream and observation. When it did not settle after a further 3-months the lesion was incised and drained under local anaesthetic. Microscopy of excised tissue showed no acid fast bacilli (AFB) present and mycobacterial cultures were negative after 12-weeks of incubation. The wound fully healed 5-weeks after the surgery without further antibiotic treatment.

One month later (September 2020) she developed a further small subcutaneous nodule (20 mm x 20 mm diameter) just medial to the index nodular lesion on the lateral aspect of the right ankle. She was diagnosed clinically with a further paradoxical reaction and commenced on prednisolone 12.5 mg daily for 2-weeks then 6.25 mg daily for one week and ceased. Despite this systemic therapy, the swelling persisted and two months after it appeared, she underwent incision and debridement of this lesion under general anaesthetic. Once again, microscopy of excised tissue showed no AFB present and mycobacterial cultures were negative after 12-weeks of incubation. The wound fully healed 3-months after the surgery without additional antibiotic treatment.

In May 2024, 4.5 years since her original BU antibiotic treatment, she again developed a painful swelling over the area of her right lateral ankle lesion. (Fig 2) It failed to resolve after 4-weeks, prompting further surgical incision and debridement under a general anaesthetic, at which time a significant amount of purulent material was found and removed. Histopathology of the excised tissue showed significant inflammation consistent with a paradoxical reaction and no AFBs present. (Fig 3) However 5–10 AFBs per 100 high power fields and 10–99 AFBs per 100 high power fields were detected on microscopy of two specimens of excised tissue, but both of these specimens were mycobacterial culture negative after 12-weeks of incubation. A *M. ulcerans* PCR of excised tissue was positive. Routine culture of the excised tissue grew no other organisms. She was managed as a late paradoxical reaction and no further antibiotic treatment was administered. Six months post-surgery the wound fully healed. No additional investigations to look for immune suppression were performed.

**Fig 2:**
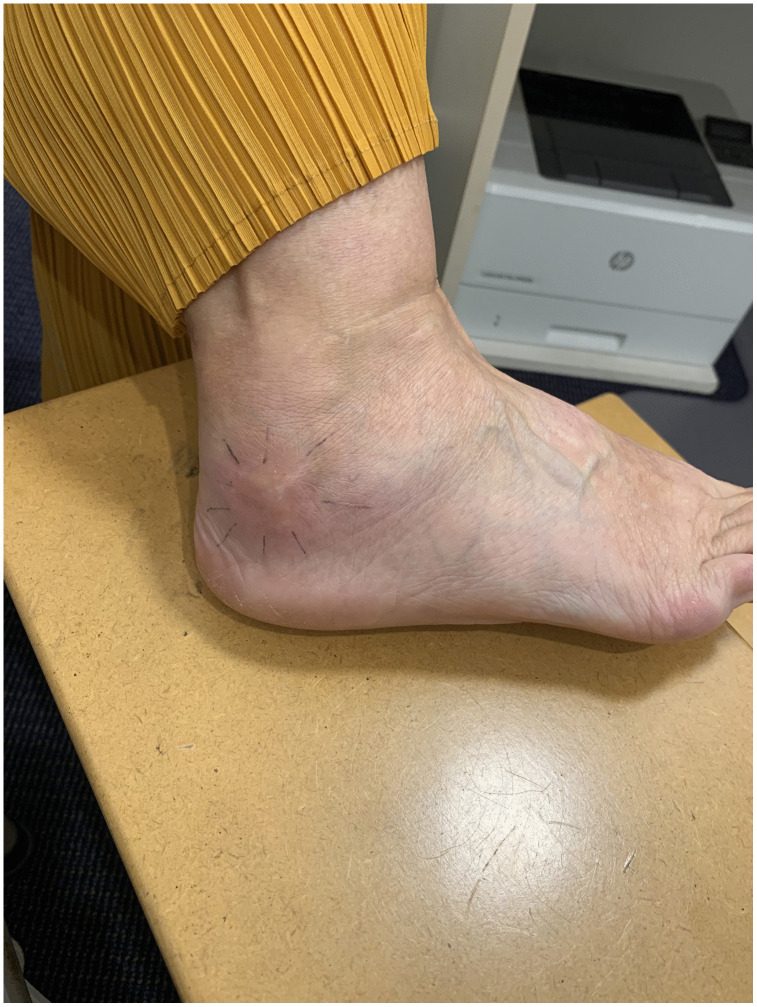
Photograph of the recurrent lesion on lateral aspect of right ankle appearing 4.5 years after initial antibiotic treatment. Skin ink markings indicate the extent of induration associated with the lesion.

**Fig 3:**
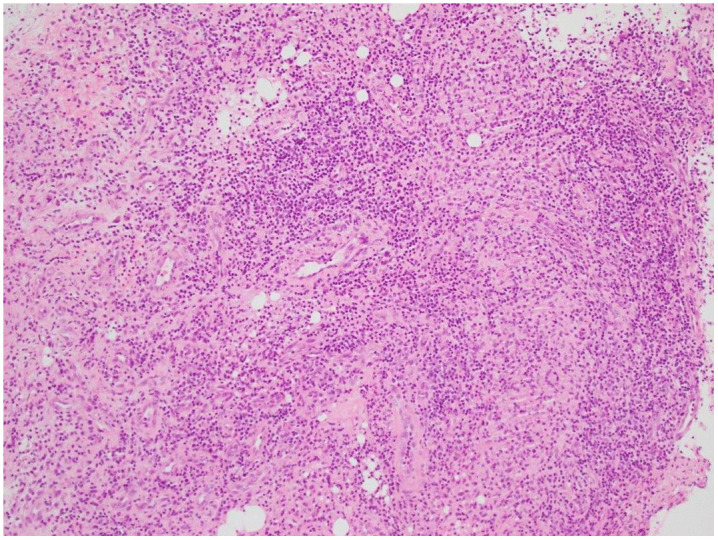
Histology of tissue from surgical debridement of recurrent right lateral ankle lesion 4.5 years after initial antibiotic therapy showing a dense mixed inflammatory cell infiltrate consistent with a paradoxical reaction.

At the time of most recent follow up, all lesions remain fully healed; 22 months following the last surgery in June 2024.

## Discussion

To our knowledge, this case represents the longest reported period (4.5 years) between initial antibiotic treatment of BU and a secondary paradoxical reaction. The previous longest period reported for a paradoxical reaction post antibiotic commencement was 409 days in a patient in Benin [1]. It is important that clinicians are aware that paradoxical reactions can occasionally be significantly delayed post antibiotic therapy to avoid unnecessary re-treatment with antibiotics which can be potentially harmful when paradoxical reactions are mistaken for active disease relapse [2].

Evidence that the recurrent lesion in 2024 was a delayed paradoxical reaction included the consistent histopathology showing intense inflammation compared with the minimal inflammation associated with disease relapse [3]. Furthermore, two mycobacterial cultures of separate specimens were culture negative, despite the presence of AFBs seen on microscopy. Finally, the lesion resolved without the use of further antibiotic treatment. It has been documented that lesions experiencing paradoxical reactions can be positive for AFB on microscopy in 59% of cases and be PCR positive for *M. ulcerans* in 88% of cases [4]. These tests indicate the presence of *M. ulcerans* DNA but not its viability. The cause of this very late paradoxical reaction was likely the ongoing presence of non-viable *M. ulcerans* DNA that had not been cleared from the lesion despite the many years that had elapsed post initial antibiotic treatment and surgery.

Paradoxical reactions are common following antibiotic treatment of BU, occurring in 20–30% of patients [4,5], as well as with treatment of other mycobacterial diseases such as tuberculosis and leprosy [6,7] With BU treatment, first paradoxical reactions usually occur not long after antibiotics are commenced with a median time to occurrence in an Australian observational cohort of 39 days (IQR 20–73 days) [4]. However, in 19% of cases, multiple paradoxical reactions can occur in the same patient, as reported in this case who had three distinct paradoxical reactions separated in time. Furthermore, 24% of paradoxical reactions occur after antibiotics have finished, as also occurred in this case [4]. The diagnosis of paradoxical reactions usually relies on the clinical picture including timing, appearance and location of lesions, and antibiotic adherence [8]. However, if in doubt, a biopsy of the lesion to identify consistent histopathology, obtain samples for mycobacterial culture and exclude alternative diagnoses may be indicated [9].

Current recommended treatment of paradoxical reactions is observation alone for mild to moderate lesions, and the use of corticosteroids or surgery in severe cases [9]. Due to ongoing pain both corticosteroids and surgery were used in this case which resulted in eventual wound healing. Importantly no further antibiotics after the initial 49-day course were required.
