# Supplementary material for: A very late paradoxical reaction occurring following antibiotic treatment of Buruli ulcer
Source: PLoS Negl Trop Dis. 2026 Jul 28;20(7):e0014585. doi: 10.1371/journal.pntd.0014585 (PMC13450833; doi:10.1371/journal.pntd.0014585)
Supplement: Supplementary file 1 [file pntd.0014585.s001.docx]

Daniel O’Brien,

Clinical Associate Professor

Department of Infectious Diseases,

Geelong Hospital, Ryrie Street,

Geelong. 3220. Australia.

Ph: +613 4215 2375

Fax: +613 4215 2385

E-mail: Danielo@BarwonHealth.org.au

To

Editors,

*PLOS Neglected Tropical Diseases.*

2/6/26

Dear Editors,

We are re-submitting our paper entitled “**A very late paradoxical reaction occurring following antibiotic treatment of Buruli ulcer**” to be considered for publication as a case report in your journal.

On the following pages please find in blue our responses to the editor’s and reviewer’s comments. We have made the relevant changes in the revised copy of the manuscript which we hope have addressed all of the concerns raised. We would be pleased to consider further revisions if needed.

All authors have seen and approved the manuscript and have contributed significantly to the work. The manuscript has not been published and is not being considered for publication elsewhere. Finally we have no conflicts of interest or funding sources to disclose.

Thank-you for considering this manuscript for publication in *PLOS Neglected Tropical Diseases*.

Yours sincerely

Daniel O’Brien

MBBS, FRACP, Dip Anat.

**Response to Editors Comments:**

This report can be considered for publication under the Symposium format but not research article format. Please consider resubmitting with that format.

We have submitted in the format of a case report as advised by Emy Janelli via email on 27/5/26.

**Journal Requirements:**

1) Please ensure that the CRediT author contributions listed for every co-author are completed accurately and in full.
At this stage, the following Authors/Authors require contributions: Christopher Mow. Please ensure that the full contributions of each author are acknowledged in the "Add/Edit/Remove Authors" section of our submission form.
The list of CRediT author contributions may be found here: [https://journals.plos.org/plosntds/s/authorship#loc-author-contributions](https://urldefense.com/v3/__https:/journals.plos.org/plosntds/s/authorship*loc-author-contributions__;Iw!!GHMCuE2BZP0!Ms0A36YibASJyh6hcwlQqEM5zcdFMNw2bfL2gEJv6JBrBny-Fi3Y92PeIuyScR6WiqdrpK2CH1UC5o65VdY$)

This has been updated as requested.

2) Please ensure that the Title in your manuscript file and the Title provided in your online submission form are the same.

This has been corrected.

3) The uploaded Manuscript file contains highlights/ tracked changes. Please provide a clean version of your manuscript from your latest manuscript version.

This has been done as requested.

4) We noticed that you used the phrase 'unpublished data' in the manuscript. We do not allow these references, as the PLOS data access policy requires that all data be either published with the manuscript or made available in a publicly accessible database. Please amend the supplementary material to include the referenced data or remove the references.

This has been removed as requested

5) Your manuscript is missing the following sections: Introduction, Results, Discussion, and Methods.  Please ensure all required sections are present and in the correct order. Make sure section heading levels are clearly indicated in the manuscript text, and limit sub-sections to 3 heading levels. An outline of the required sections can be consulted in our submission guidelines here:
[https://journals.plos.org/plosntds/s/submission-guidelines#loc-parts-of-a-submission](https://urldefense.com/v3/__https:/journals.plos.org/plosntds/s/submission-guidelines*loc-parts-of-a-submission__;Iw!!GHMCuE2BZP0!Ms0A36YibASJyh6hcwlQqEM5zcdFMNw2bfL2gEJv6JBrBny-Fi3Y92PeIuyScR6WiqdrpK2CH1UCNTOPM7g$)

We have submitted in the format of a case report as advised by Emy Janelli via email on 27/5/26.

6) Please insert an Ethics Statement at the beginning of your Methods section, under a subheading 'Ethics Statement'. It must include:
i) The full name(s) of the Institutional Review Board(s) or Ethics Committee(s)
ii) The approval number(s), or a statement that approval was granted by the named board(s)
iii) A statement that formal consent was obtained (must state whether verbal/written) OR the reason consent was not obtained (e.g. anonymity). NOTE: If child participants, the statement must declare that formal consent was obtained from the parent/guardian.].

This has been added as requested.

7) Please upload all main figures as separate Figure files in .tif or .eps format. For more information about how to convert and format your figure files please see our guidelines: 
[https://journals.plos.org/plosntds/s/figures](https://urldefense.com/v3/__https:/journals.plos.org/plosntds/s/figures__;!!GHMCuE2BZP0!Ms0A36YibASJyh6hcwlQqEM5zcdFMNw2bfL2gEJv6JBrBny-Fi3Y92PeIuyScR6WiqdrpK2CH1UC0oGwS7o$)

This has been done as requested.

8) We note that your Data Availability Statement is currently as follows: "No dataset was used in this research". Please confirm at this time whether or not your submission contains all raw data required to replicate the results of your study. Authors must share the “minimal data set” for their submission. PLOS defines the minimal data set to consist of the data required to replicate all study findings reported in the article, as well as related metadata and methods ([https://journals.plos.org/plosone/s/data-availability#loc-minimal-data-set-definition](https://urldefense.com/v3/__https:/journals.plos.org/plosone/s/data-availability*loc-minimal-data-set-definition__;Iw!!GHMCuE2BZP0!Ms0A36YibASJyh6hcwlQqEM5zcdFMNw2bfL2gEJv6JBrBny-Fi3Y92PeIuyScR6WiqdrpK2CH1UCN82dKk4$)).

We confirm the submission contains all of the data required to replicated all study findings reported in the article.

9) Kindly amend your competing statement in the online submission form to align with the journal's style guidelines: 'The authors declare that there are no competing interests.'

This has been corrected as requested.

**Response to Reviewers' Comments:**

**Methods**

Reviewer #1: The manuscript is a well written case report with an interesting discussion and contributes to our understanding of sterile inflammatory reactions in BU with practical management implications and also implications for our understanding of immunopathogenesis.

**Results**


Reviewer #1: It is well written, easily followed and the figure is appropriate.

**Conclusions**

Reviewer #1: The discussion and conclusion is appropriate and supported by the data presented.

**Editorial and Data Presentation Modifications?**

Reviewer #1: Line 88-89: Change to: "However, if in doubt, biopsy of the lesion to identify consistent histopathology, obtain samples for mycobacterial culture and exclude alternative diagnoses may be indicated."

Thanks for this useful suggestion – we have changed manuscript as suggested.

Please indicate if the patient has any significant past medical history, in particular immunosuppression that might have influenced the initial presentation of multiple lesions or the subsequent course of recurrent sterile inflammatory lesions.

This has been added in line 25.

Was additional immunosuppression screen performed?

This has been added in lines 59-60.

**Summary and General Comments**

Reviewer #1: I think that this is a well written case report and adds to the spectrum of paradoxical reactions seen in BU. I think it is of significant interest to clinicians treating BU, with practical implications, and also to those who manage other mycobacterial infections where paradoxical reactions are problematic.

We thank the reviewer for these comments.
