# Supplementary material for: A very late paradoxical reaction occurring following antibiotic treatment of Buruli ulcer
Source: PLoS Negl Trop Dis. 2026 Jul 28;20(7):e0014585. doi: 10.1371/journal.pntd.0014585 (PMC13450833; doi:10.1371/journal.pntd.0014585)
Supplement: Supplementary file 2 [file pntd.0014585.s002.docx]

Daniel O’Brien,

Clinical Associate Professor

Department of Infectious Diseases,

Geelong Hospital, Ryrie Street,

Geelong. 3220. Australia.

Ph: +613 4215 2375

Fax: +613 4215 2385

E-mail: Danielo@BarwonHealth.org.au

To

Editors,

*PLOS Neglected Tropical Diseases.*

22/6/26

Dear Editors,

We are re-submitting our paper entitled “**A very late paradoxical reaction occurring following antibiotic treatment of Buruli ulcer**” to be considered for publication as a case report in your journal.

On the following pages please find in blue our responses to the editor’s and reviewer’s comments. We have made the relevant changes in the revised copy of the manuscript which we hope have addressed all of the concerns raised. We would be pleased to consider further revisions if needed.

All authors have seen and approved the manuscript and have contributed significantly to the work. The manuscript has not been published and is not being considered for publication elsewhere. Finally, we have no conflicts of interest or funding sources to disclose.

Thank-you for considering this manuscript for publication in *PLOS Neglected Tropical Diseases*.

Yours sincerely

Daniel O’Brien

MBBS, FRACP, DMedSc, Dip Anat.

**Response to Editors Comments:**

Your manuscript is missing the following sections: Introduction, Results, and Methods.  Please ensure all required sections are present and in the correct order. Make sure section heading levels are clearly indicated in the manuscript text, and limit sub-sections to 3 heading levels.

We have included the section headings as requested (introduction, methods and results).

**Response to Reviewers' Comments:**

**Editorial and Data Presentation Modifications?**

Reviewer #2:

I think a clinical photograph of the late presentation could be a useful addition.

Thanks for this suggestion. We have now added a photograph of the initial BU lesions (figure 1) as well as a photograph of the late presentation (figure 2). In the process, the original figure 1 from previous manuscript has now been updated to figure 3.

Were there any other cultures that were positive (eg for S. aureus)?

No other organisms were grown from routine cultures of the excised tissue. This information has now been added to the manuscript.

The original diagnostic specimen was PCR positive but "not cultured". The reference laboratory that performs the PCRs in Victoria routinely cultures all first PCR requests so may have this information if asked. If they do, it would be worth reporting. However I have no doubt the original diagnosis based on PCR alone is correct in this clinical setting.

The original PCR swab specimen was not sent to the Victorian Reference Laboratory and therefore was not cultured.
